# Coupling Relationship of Leaf Economic and Hydraulic Traits of *Alhagi* *sparsifolia* Shap. in a Hyper-Arid Desert Ecosystem

**DOI:** 10.3390/plants10091867

**Published:** 2021-09-09

**Authors:** Hui Yin, Akash Tariq, Bo Zhang, Guanghui Lv, Fanjiang Zeng, Corina Graciano, Mauro Santos, Zhihao Zhang, Peng Wang, Shuyong Mu

**Affiliations:** 1 State Key Laboratory of Desert and Oasis Ecology, Xinjiang Institute of Ecology and Geography, Chinese Academy of Sciences, Urumqi 830011, China; yinhui@ms.xjb.ac.cn (H.Y.); zhangbo@ms.xjb.ac.cn (B.Z.); zhangzhihao9211@163.com (Z.Z.); wangp@ms.xjb.ac.cn (P.W.); symo@ms.xjb.ac.cn (S.M.); 2 College of Resource and Environment Sciences, Xinjiang University, Urumqi 830046, China; ler@xju.edu.cn; 3 University of Chinese Academy of Sciences, Beijing 100049, China; 4 Xinjiang Key Laboratory of Desert Plant Roots Ecology and Vegetation Restoration, Xinjiang Institute of Ecology and Geography, Chinese Academy of Sciences, Urumqi 830011, China; 5 Cele National Station of Observation and Research for Desert-Grassland Ecosystems, Cele 848300, China; 6 Institute of Plant Physiology, National Council for Scientific and Technical Research, National University of La Plata, La Plata 1900, Argentina; corinagraciano@gmail.com; 7 Laboratory of Plant Physiology, Department of Botany, Federal University of Pernambuco, Recife 50670-901, Brazil; mauro.gsantos@ufpe.br

**Keywords:** coupling relation, leaf mass per area, mesophyll structure, stomata traits, tissue and vein density

## Abstract

In this study, *Alhagi* *sparsifolia* Shap. was used to test the hypothesis that leaf economic and hydraulic traits are coupled in plants in a hyper-arid region. Five economic traits and six hydraulic traits were examined to explore the relationship. Results showed that the stomatal density (SD) on both surfaces was coupled with maximum stomatal conductance to water vapor (g_wmax_) and leaf tissue density (TD). SD on adaxial surface (SD_aba_) was significantly positively related to vein density (VD) but negatively related to leaf thickness (LT) and stomatal length on adaxial surface (SL_ada_). Nitrogen concentration based on mass (N_mass_) was significantly negatively correlated with leaf mass per area (LMA), LT, and VD, whereas nitrogen concentration based on area (N_area_) was significantly positively related to LMA and TD. Mean annual precipitation (MAP) contributed the most to the changes in LT and stomatal length (SL). Soil salt contributed the most to TD, SD, and g_wmax_. Soli nutrients influenced the most of LMA and VD. Mean annual temperature contributed the most to N_mass_ and N_area_. In conclusion, the economics of leaves coupled with their hydraulic traits provides an economical and efficient strategy to adapt to the harsh environment in hyper-arid regions.

## 1. Introduction

Leaf functional traits are highly profiled in ecology because of their closely related to plants’ growth and adaptation to environmental stress [1,2,3]. The “fast slow” economic spectrum widely exists in plant communities, that is, slow-growing species have a high tissue density, a low resource acquisition rate, and a high construction cost. In resource-poor environments, these slow species often form the dominant species in resource-poor environments [1,2]. Leaf functional traits, such as leaf mass per area (LMA), nitrogen concentration based on mass (N_mass_) and area (N_area_), leaf tissue density (TD), and leaf thickness (LT), are highly associated with photosynthesis capacity. These traits always vary along a continuous spectrum and can reflect the quick to slow ecological strategies relative to leaf investment and photosynthetic payback, which are collectively referred to ‘economic traits’ [1,2,3,4]. However, using only a single axis of economic traits is insufficient to explain plant survival strategies, especially in arid areas, where water deficit may affect plant survival. Other traits, such as vein density (VD), stomatal density (SD), stomatal length (SL), and maximum stomatal conductance to water vapor (g_wmax_), reflect water demand and supply balance and are accordingly defined as “leaf hydraulic traits” [3,5]. Both economic and hydraulic traits can indicate the trade-off between plant production and investment. Thus, exploring the correlation between leaf economic and hydraulic traits is crucial to reveal the mechanism of plant water carbon balance and expanding plant traits spectrum [2,4,6,7,8].

Despite years of research, scientists have not yet reached a consensus on whether a coupling relationship exists between economic and hydraulic traits [3,4,9,10,11]. Many studies suggest that economic and hydraulic traits should be coupled because the stomata are the common pathway for CO_2_ uptake and water loss [9,12,13]. In particular, carbon starvation caused by stomatal closure is an important cause of plant death under prolonged extreme drought conditions [14]. In arid environments, both VD and SD are high. High VD and SD are important adaptive strategies for maintaining an appropriate mesophyll space between terminal veins and stomata to balance carbon absorption and water transpiration [15,16,17]. For example, in 33 perennial shrubs native to the arid valley of southwest China, VD and SD were found to be coupled well with light-saturated photosynthetic rates, thereby enabling these plants to maintain a robust balance between water loss and carbon absorption [18]. In semi-arid regions, the economic traits—LMA, LT, TD, N_mass_, N_area_, and the hydraulic traits—VD, SD, SL, g_wmax_ were found to be coupled across 47 woody species [4]. By comparing the same traits with plants in humid regions [3], Yin et al. inferred that this coupling relation is caused by low water availability [4], and they further suggested that the correlation between stomatal and leaf vein traits seems to be the key link between economic and hydraulic traits under drought conditions. By contrast, the economic and hydraulic traits in humid environments seem to undergo decoupling. In tropical–subtropical forests with adequate water resources, the economic and hydraulic traits of plants are decoupled to acquire multiple trait combinations to be able to utilize more resources [3,4,19]. Using large data sets, Sack et al. also found that LMA is not associated with VD [10]. Although VD is independent of LMA, it strongly affects hydraulic conductivity, thereby affecting stomatal conductance and photosynthetic rate [17]. When a sufficient water supply is available, most plants are anisohydric, that is, their stomata keep remain open even when water potential is reduced [20,21,22]. In comparison to cases of interannual or seasonal variations, the hydraulic behavior of the same species may change from anisohydry to isohydryby closing their stomata at noon to maintain the minimum water potential [23,24]. Therefore, we speculated that stomatal regulation under dry conditions might affect carbon water coupling.

In hyper-arid ecosystems, high temperature, low precipitation, nutrient limitation, and salinity stress lead to low species diversity [25]. Plants adapt to such a harsh environment through physiological and morphological adaptations, including changes in leaf anatomy, economic traits, and hydraulic traits [26,27,28]. *Alhagi* *sparsifolia* Shap. (Fabales: Fabaceae), which widely distributed in hyper-arid regions across the middle and eastern Asia, is one such species. Owing to the heterogeneity of climatic variations, soil water supply, and nutrients availability in the distributional area of *A. sparsifolia*, this plant is evidently different among sites [29], with corresponding leaf morphological differences [30]. The leaves of *A. sparsifolia* are amphistomatous, with stomata on both the upper and lower surfaces [31]. Previous studies have shown that compared with hypostomatous leaves with stomata only on the lower surface, amphistomatous leaves can considerably reduce the transport distance of CO_2_ and H_2_O in mesophyll tissues and enhance the gas exchange capacity between the mesophyll and atmosphere [32]. Moreover, *A.* *sparsifolia* has isobilateral leaves, palisade parenchyma neatly are ranged in the upper and lower epidermis, and the sponge parenchyma only distributes in the center of the mesophyll [31]. Increasing the ratio of palisade parenchyma to sponge parenchyma (PT/ST) is a vital life strategy for plants to cope with dry environments [33,34], and the trade-off between PT and ST may lead to the correlation between leaf economic and hydraulic traits in the plants in semi-arid regions [4]. However, the issue of whether changes in the anatomical structure of isobilateral leaves are related to the economic and hydraulic traits is still unclear.

The roots length of *A. sparsifolia* can reach at the depth of 12 m or even 30 m [30]. The developed roots can connect with groundwater, and then hydraulic lift occurs [35]: the deep roots absorb water at night and move it to the upper soil profile, where it is stored until it is absorbed the next day [36,37,38]. The aboveground part of *A. sparsifolia* does not suffer from water deficit during the growing season [7,30], indicating that this species can balance the relationship between material investment in water transportation and water loss in transpiration [39]. However, short-term flood or irrigation in summer can increase the leaf area of *A. sparsifolia* by 2.76 times [39]. Therefore, we speculated that in different regions, the upper soil water content may also affect the functional traits of *A. sparsifolia*. As in many hyper-arid regions worldwide, the desert ecosystems in northwest China are being affected by extensive salinization [40]. Salinity reduces the soil water potential as a form of drought, rendering the absorption of water from the soil by plants more difficult [41,42,43,44]. Furthermore, salinity affects soil pH value and then influences the absorption of nutrients by plants [45], thus negatively affecting water supply and photosynthesis. *A. sparsifolia* is a xero-halophyte with a high tolerance to drought and salt stress [46,47,48]. Thus, its functional traits should be adapted functional traits to drought and salinity. As a member of family Fabaceae, *A. sparsifolia* can establish a symbiotic relationship with nitrogen-fixing bacteria in the soil, capturing N_2_ from the atmosphere and making it available to plant root in exchange for carbohydrate exudates [49,50]. This mechanism is an important survival strategy for *A. sparsifolia* to overcome environments with a low nitrogen supply [49,50]. However, under water and salinity stress conditions, the amount of carbon may be insufficient to invest in root exudates and biological nitrogen fixation (BNF) [50]. Groundwater and soil nitrogen are important nitrogen sources affecting nitrogen absorption [50], nitrogen concentration, and photosynthesis in leaves. Specifically, we hypothesized that a coupling relationship exists between the leaf economic and hydraulic traits of *A. sparsifolia* in hyper-arid regions, and this relationship is closely related to mesophyll structure. Moreover, we postulated that the leaf economic and hydraulic traits of *A. Sparsifolia* change as an essential strategy to respond to different stress conditions involving climatic variations, soil water supply, nutrients availability, and salinity in hyper-arid ecosystems.

## 2. Materials and Methods

### 2.1. Sampling Sites

**Figure 1:**
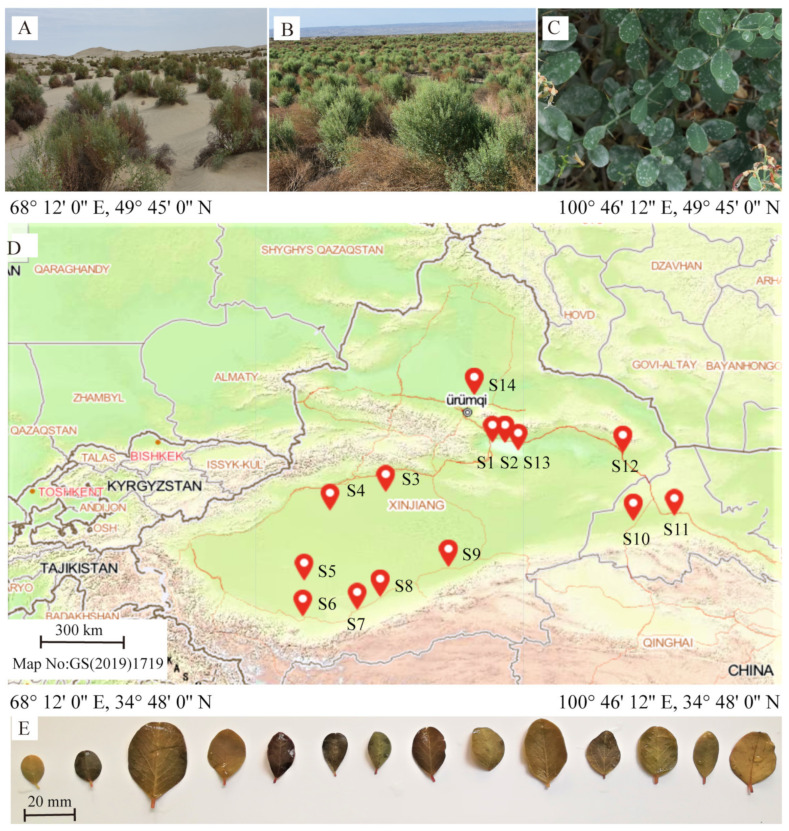
Geographic location of the sampling sites and growing conditions of *Alhagi* *sparsifolia*. (**A**,**B**) Photographs of *A. sparsifolia* populations showing the typical growth conditions at two sampling sites, S9 and S13. (**C**) The leaves of *A. sparsifolia*. (**D**) The geographic location of sampling sites, marked as S1–S14. (**E**) The leaf photos are arranged left to right by sampling site, from S1 through S14.

*A. sparsifolia* is a widely distributed plant found in the hyper-arid regions of northwest China. This study was conducted from July 2018 to August 2018. To expand the heterogeneity of environmental conditions, we selected 14 sampling sites along a longitude ranging from 72°41′24″ E to 99°41′24″ E, and latitude ranging from 35°34′11″ N to 48°7′48″ N (Figure 1). Three 10 m × 10 m plots were randomly set at each sampling site. We obtained precipitation and temperature data from the “WorldClim version 2.1” climate data for 1970–2000 (the spatial resolution was 30 s) [51] by using the R 4.2.0 software. The mean annual precipitation (MAP) of the study location ranged from 16mm to 166 mm.

### 2.2. Measurement of Traits

#### 2.2.1. Economic Traits

In each plot, we randomly chose 30 leaves from at least 10 *A. sparsifolia* individuals to measure leaf mass per area (LMA) and leaf thickness (LT). From at least 20 different individuals, we collected approximately 10 g mixture of fully expanded leaves from each plot to determine nitrogen concentration per mass (N_mass_). We pressed leaves of plants sampled from each plot with a glass. Using a ruler as a scale, we took images of the leaves, and measured the area of each blade by using the Image J software (NIH Image, Bethesda, MD, USA). To calculate LMA, we used the sum value of 30 leaves per plot as the total leaf area. All leaves collected from each plot were dried at 80 °C for 48 h and then weighed to determine dry leaf biomass [49]. LMA was calculated as dry leaf biomass divided by total leaf area [4]. We used the same leaves to calculate LT by using a digital caliper at an intermediate position between the leaf margin and the midrib for each leaf [52]. We then used the average value of each plot as LT. Leaf tissue density was calculated by dividing LMA with LT [3,4]. We measured the nitrogen concentration (N_mass_) of the leaves via the Kjeldahl method (FOSS Kjeltec 8400, FOSS, Hoganas, Sweden), whereby nitrogen concentration per area (N_area_) was calculated as the product of N_mass_ and LMA [3].

#### 2.2.2. Hydraulic Traits

We collected another set of 30 leaves from at least 10 individuals per plot and immediately placed them in FAA fixing solution for the subsequent determination of hydraulic traits. Specifically, we removed three leaves from the FAA solution and then transferred them into a 15% NaOH solution to degrade their mesophyll cells. Afterward, we bleached the leaves in 10% H_2_O_2_ solution. We stained the leaves with 1% safranine O, and then photographed three fields of veins at 100×magnification under an optical microscope (Olympus DSC-600, Olympus, Tokyo, Japan) while avoiding the mid-central vein. Total vein length was calculated as the full vein length per unit leaf area [9]. The average value of each plot was used for further analysis.

We analyzed the stomatal density (SD) and stomatal length (SL) ofthe leaves via scanning electron microscope (SEM) (Zeiss Supra55 VP, Carl Zeiss, Oberkochen, Germany) after dehydrating the sampled leaves in graded ethanol series (70%, 80%, 85%, 90%, 95%, and 100% twice, with each series lasting for 15 min). Subsequently, ethanol was replaced with 2-Methyl-2-propanol, and then the leaves were desiccated in a freeze dryer (Christ-ALPHA 2-4 LSCplus, Martin Christ, Osterode am Harz, Germany). The leaves were stuck to the sample holders, placed in the ion sputtering instrument (Hitachi E-1045, Hitachi, Tokyo, Japan), and sprayed with platinum for 2 min. We removed three leaves from each plot and took 10 photographs of these leaves at 200× magnification to calculate SD and SL, using the derivations:

- SD_ada_, stomata number per area on the adaxial surface of a leaf;
- SD_aba_, stomata number per area on the abaxial surface of a leaf;
- SL_ada_, the length of the guard cell on the adaxial surface of a leaf;
- SL_aba_: the length of the guard cell on the abaxial surface of a leaf.

g_wmax_ was calculated follows:

(1) $$g_{w\max}=d\alpha LD/(\nu (0.5+0.627\sqrt{\alpha })),$$

where *d* is the diffusivity of water in the air, *L* denotes SL, and *D* represents SD. Given that the stomata exist on both leaf surfaces, SD was calculated here as the sum of SD_ada_ and SD_aba_, whereas SL was calculated as the mean of SL_ada_ and SL_aba_. Furthermore, *ν* represents the molar volume of air, and α represents a fraction of stomatal size to the mean maximum stomatal pore area; a mid-range value of 0.12 for α was used in accordance with previous studies [3,4,53].

#### 2.2.3. Mesophyll Structure

Three leaves from each plot were removed from the FAA solution and then dehydrated with ethanol series (70%, 80%, 85%, 90%, 95%, 100% twice, each series lasting for 15 min). Subsequently, ethanol was replaced with acetone and paraffin. The leaves were then embedded in paraffin for their sectioning; the transverse sections were cut into 10–20 μm slices witha rotary microtome (Leica RM2235, Leica Biosystems, Nussloch, Germany) and then mounted on glass slides. The slices were stained with safranin O and counterstained by fast green. Afterward, which micrographs were taken under an optical microscope (Olympus DSC-600, Olympus, Tokyo, Japan). For each section, five measurements of palisade parenchyma thickness in the upper layer (UPT) and the lower layer (LPT), as well as spongy parenchyma thickness (ST) were measured using Image J software (NIH Image, Bethesda, MD, USA). PT was calculated by calculating the sum of UPT and LPT, and then we calculated the ratio of PT/ST. The average values of the three leaves in each plot were taken as the value of this plot.

#### 2.2.4. Soil Properties

In each plot, we collected three soil samples were collected with a 1 m soil profile at depths of 0–20, 20–40, 40–60, 60–80, and 80–100 cm. We dried approximately100 g of the soil samples in an oven to determine the soil water content (SWC). Subsequently, we air-dried, grounded, and passed through a 100-mesh sieve about 500 g soil samples to evaluate soil alkali-hydrolysable nitrogen (SAN) and soil available phosphorus (SAP), soil salt content (SS), and soil pH value (pH). We ground the soil to a fine powder by using a ball mill (MM400, Retsch, Haan, Germany) to determine soil organic carbon (SOC) [54]. We measured SS via the oven drying method after water was extracted with a 1:5ratios of soil: deionized water [54]. We evaluated SAN via the alkali solution diffusion method as described by Bao [54]. SAP was extracted with 0.5 mol/L NaHCO_3_ solution and evaluated by using the molybdate/ascorbic acid blue method [54]. Soil samples were digested in the K_2_Cr_2_O_7_ H_2_SO_4_ solution for 5 min and measured via the titration method [54] to assess SOC. Soil pH was measured with a pH meter (SevenEasy pH, Mettler-Toledo, Schwerzenbach, Switzerland) after water extracting using a soil: deionized water ratio of 1:2.5 [54]. The mean values of SWC, SS, SAN, SAP, SOC, and pH within 1 m was used for further analysis.

### 2.3. Statistical Analysis

We calculated the means, standard errors (SE), and coefficient of variation (CV) of each trait using all of the values for all plots. After the data were standardized, the “vegan” package in R 4.0.2 (R Development Core Team) was used to analyze the multivariate association of the whole set of economic and hydraulic characteristics through principal component analysis (PCA). From these results, the loading scores for each functional trait along the first four axes of the PCA were obtained and displayed by the “vegan” package in R 4.0.2. Canonical correlation analysis (CAA) was carried out [55] with SPSSAU (22.0) online application software [56] to verify the coupling relationship between the groups of economic and hydraulic traits. The “corrplot” package in R 4.0.2 was applied to perform Pearson correlation on the entire dataset for the pairwise analysis of the relationship between plant functional traits. The “vegan” package in R 4.0.2 was used to conduct linear regression on the whole data set to express the relationship of functional traits with MAP, SWC, and mesophyll structure. Here, log10 transformation was applied onthe data to increase their linear relationship. We used the generalized linear model of the “glm” package in R 4.0.2 to select the environmental variables (MAP, MAT, SWC, SS, SAN, SAP, SOC, and pH) that affected each functional trait. All of the data log10 transformed to increase their normality. The model with a lower AIC value was selected for importance analysis. We utilized the “relaimpo” package in R 4.0.2 to perform important relative analysis and selected the “lmg” metric to calculate the contributions of the relevant factors to each trait.

## 3. Results

### 3.1. Variation in Leaf Functional Traits

Among all the traits, SL_ada_ and SL_aba_ and VD showed minor variation across sampling sites; the CV values of SL_ada_, SL_aba_, and VD were11.97%, 10.12%, and 12.46%, respectively. The other traits had high CV values that reached nearly 20% (Table 1). Mean and SE values for traits measured for each sampling site showed in the Table S1.

### 3.2. Relationships among Leaf Economic Traits, Hydraulic Traits, and Mesophyll Structure

**Figure 2:**
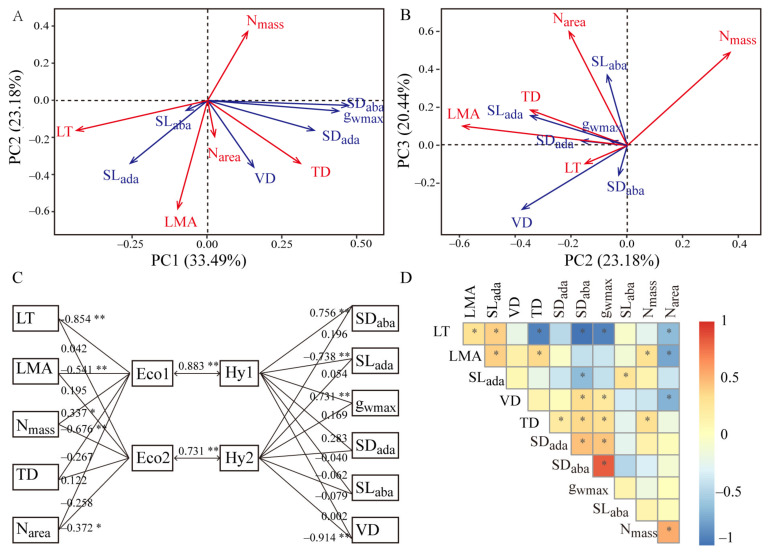
The relationship between economic and hydraulic traits. (**A**,**B**) Principal component analysis (PCA) showing the leaf economics traits (red lines) and leaf hydraulic traits (blue lines) coupled along the different axis. The parenthesized numbers on each axis are the percentage of total variation explained by that given axis component. (**C**) Canonical correlation analysis to test the coupling relationship between the groups of economic and hydraulic traits. The numbers beside each trait show the correlation coefficient of each trait to canonical variance. Eco1 and Eco2 are the first two canonical variances in the economic traits group, whereas Hy1 and Hy2 are the first two canonical variances in the hydraulic traits group. The numbers between Eco1 and Hy1, Eco2 and Hy2 are their correlation coefficient. * marked as *p* < 0.05, ** marked as *p* < 0.01. (**D**) Relationship between pairwise plant functional traits. * marked as *p* < 0.05. The abbreviations of leaf economic and hydraulic traits are shown in Table 1.

PCA revealed that the economic and hydraulic traits of *A. sparsifolia* were not orthogonal but rather coupled along PC axes. The first PC axis (PC1) accounted for 33.25% of the total variation. Notably, SD_ada_, SD_aba_, g_wmax_, TD, LT, and VD provided the highest contributions to PC1. However, LT contributed inversely to variation vis-à-vis other leaf traits. The second PC axis (PC2) accounted for 20.37% of the total variation; LMA, TD, N_mass_, VD, and SL_ada_ had the highest contributions to PC2, with N_mass_ contributing in the opposite direction as the other traits (Figure 2A, Table S2). PC3 explained 18.22% of the total variation, with N_area,_ N_mass_, SL_aba_, and VD presenting the highest contributions (Figure 2B).

As shown in Figure 2C, LT, LMA, N_mass_, SD_aba_, SL_ada_, g_wmax_ contributed significantly to the first pair of canonical variance extracted by CAA (Eco1 and Hy1). By contrast, N_mass_, N_area_, and VD had the highest contributions to the second pair of canonical variance (Eco2 and Hy2). The correlation coefficients of the first two pairs of canonical variance were 0.883 and 0.731, illustrated that the group of economic traits had a significant positive correlation with hydraulic traits.

SD_ada_ and SD_aba_ showed a significantly positive relationship with g_wmax_ and TD. SD_aba_ but not SD_ada_ showed a significant positive correlation with VD and a significantly negative relationship with LT and SL_ada_. N_mass_ demonstrated a significantly negative relationship with LMA, LT, and VD, whereas N_area_ exhibited a significantly positive relationship with LMA and TD (Figure 2D).

As can be seen from Table 2, UPT was significantly positively related to LMA, LT, and SL_ada_, and negatively correlated with TD, g_wmax_ and SD_aba_. LPT had no relationship with SL and SD. ST was negatively correlated with TD, SD, and g_wmax,_ and PT/ST was positively correlated to VD and SD.

### 3.3. Contributions of the Environmental Variables on Leaf Functional Traits

**Figure 3:**
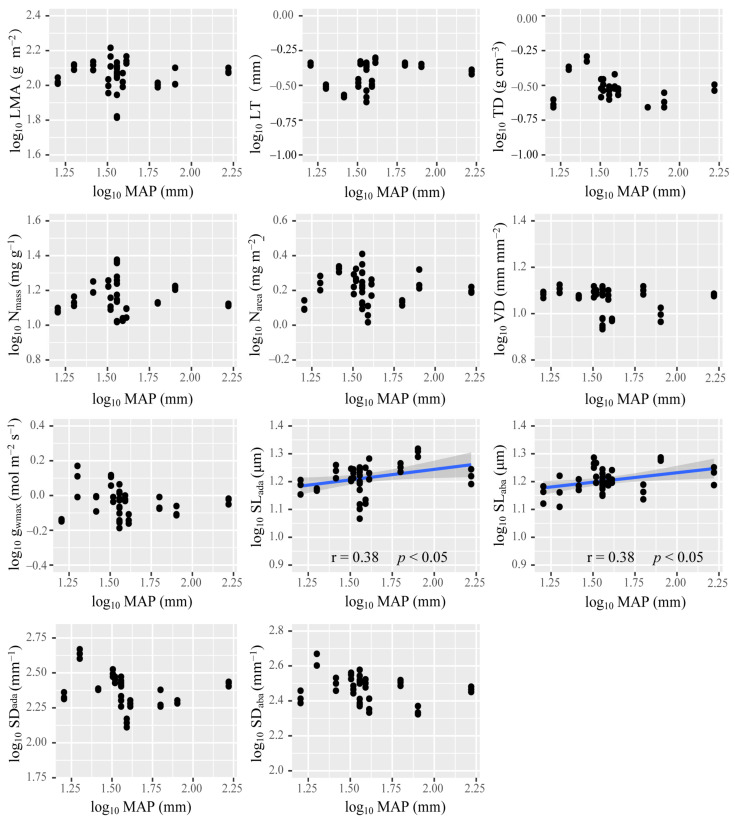
Relationship of log_10_ MAP with functional traits. Linear correlations are indicated as blue lines (having a *p*-value < 0.05). Shaded bands around each line represent the 95% confidence interval for the linear regression. MAP: mean annual precipitation. The abbreviations of leaf economic and hydraulic traits are shown in Table 1.

**Figure 4:**
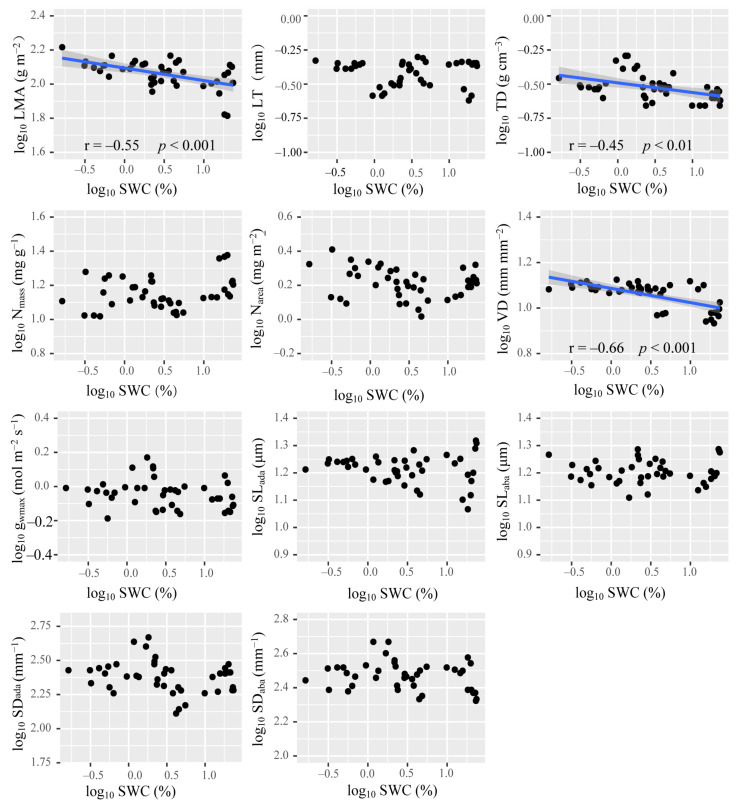
Relationship of log_10_ SWC with leaf functional traits. Linear correlations are indicated as blue lines (having a *p*-value <0.05). Shaded bands around each line represent the 95% confidence interval for the linear regression, SWC: soil water content. The abbreviations of leaf economic and hydraulic traits are shown in Table 1.

**Figure 5:**
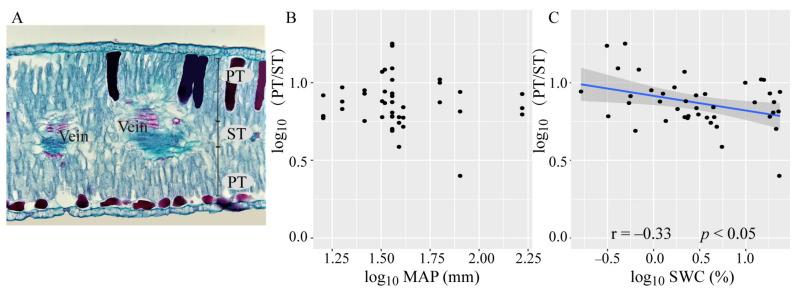
Antomical structure of *A*. *sparsifolia* and the relationship of PT/ST with MAP and SWC. (**A**) the transverse leaf sections of *A*. *sparsifolia*. (**B**) the relationships between PT/ST and MAP. (**C**) the relationships between PT/ST and SWC. Linear correlations are indicated as blue lines (having a *p*-value < 0.05). Shaded bands around each line represent the 95% confidence interval for the linear regression. PT, thickness of palisade parenchyma; ST, thickness of spongy parenchyma; PT/ST, ratio of thickness of palisade parenchyma to spongy parenchyma; MAP, mean annual precipitation; SWC, soil water content.

**Figure 6:**
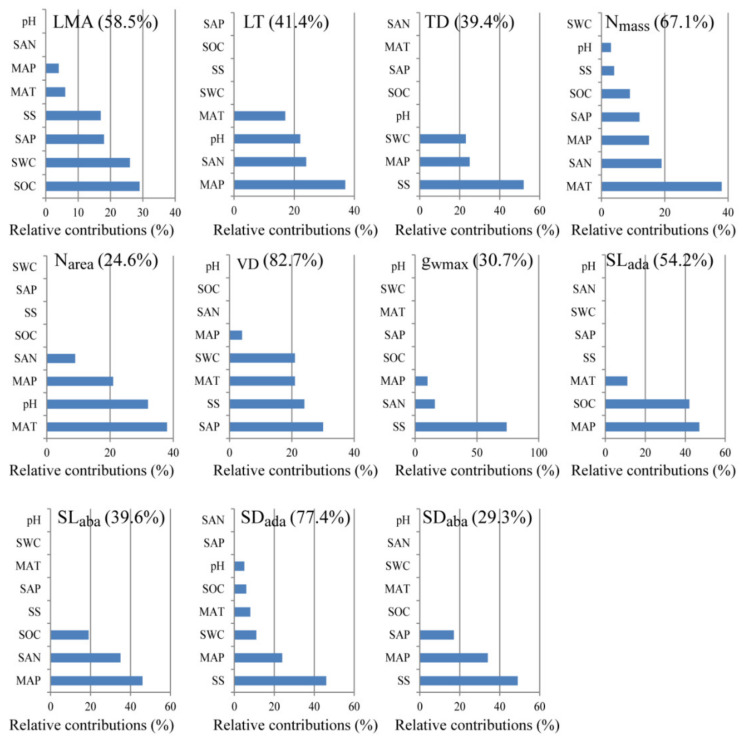
Relative contributions of environmental variables on each leaf functional trait. The numbers in brackets indicate the contribution of all environmental variables to this trait. MAP, mean annual precipitation; MAT, mean annual temperature; pH, soil pH value; SAN, soil alkali-hydrolyzable nitrogen; SAP, soil available phosphorus; SOC, soil organic carbon, SS, soil total salt content; SWC, soil water content. The abbreviations of leaf economic and hydraulic traits are shown in Table 1.

Among all the functional traits investigated in this work, only SL_ada_ and SL_aba_ was positively correlated with MAP (Figure 3). LMA, TD, and VD were negatively correlated with SWC (Figure 4). Nevertheless, SWC rather than MAP, was the most important driver ofthe changed in the PT/ST of *A. sparsifolia* (Figure 5). Relatively important analysis (Figure 6) revealed that MAP and soil nutrients contributed significantly to the changes in LT and SL. SS provided high contributions to SD and g_wmax_. SWC, SOC, SAP, and SS contributed greatly to LMA, TD, and VD. Mean annual temperature (MAT) along with pH and MAP, had high contributions to N_mass_ and N_area_.

## 4. Discussion

Plants can exhibit variation in their functional traits and their combinations as a mechanism for adaptation to different environmental conditions [57,58]. In this study, the CV of most functional traits was approximately 20% (Table 1). This result suggested that under the influence of different environmental variables, the functional traits of *A. sparsifolia* could exhibit high variation.

### 4.1. Coupling Relationship between Leaf Economic and Hydraulic Traits Based on the Mesophyll Structure

**Figure 7:**
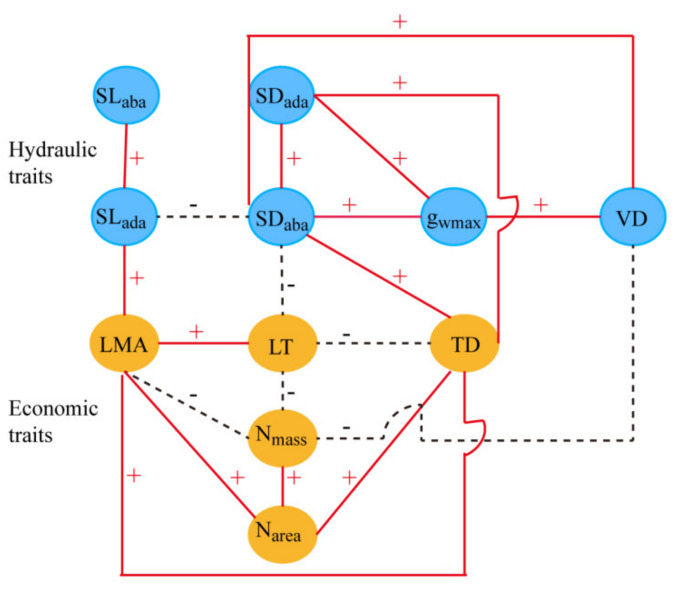
Network diagram of the correlations among leaf hydraulic and economic traits of *A. sparsifolia*. Red lines and black dotted lines indicate positive and negative correlations, respectively. The abbreviations of leaf economic and hydraulic traits are shown in Table 1.

In terrestrial plants, the maintenance of the balance between CO_2_ uptake and H_2_O transpiration is the major biophysical constraint of carbon gain and survival [4,12,59]. Franks and Farquhar [60] found that compared with C4 plants, most C3 plants need to maintain higher stomatal conductance to provide sufficient CO_2_ for photosynthesis. At the same time, C3 plants need to tolerate high atmospheric vapor pressure (VPD), and may be threatened by increased transpiration water loss. *A. sparsifolia* is an isohydric plant [61] that can maintain a constantly midday leaf water potential by reducing stomatal conductance as to limit transpiration. The maximum stomatal conductance forCO_2_ and H_2_O depends on SD and SL [53]. Under dry conditions, small and dense stomata are advantageous because tiny pores are more flexible than large ones. However, the increase in SD requires considerably more material investment than the increase in SL [53]. Therefore, the trade-off between SD and SL is an important economic strategy for plants. In this study, stomata were found to be located on the upper and lower surfaces of the leaves of *A. sparsifoila*. SD on both sides of the blade was significantly positively correlated with g_wmax_ (Figure 7). By contrast, SL lacked a relationship with g_wmax_, indicating that g_wmax_ was mainly determined by SD. The significant coupling always exists between SD and VD [15], enables plants to maintain a constantly water potential when stomata are opened. The main characteristic of deep-rooted desert plants is that their roots are connected to groundwater and form developed hydraulic channels for water transport to the aboveground parts for plant growth. Therefore, overcoming hydraulic resistance and ensuring leaf water supply are essential for the survival of plants in hyper-arid regions.

Leaf resistance to water transport accounts for 30% of the whole plant, and the higher resistance that occurs in the leaf comes from the outside-xylem pathway [62]. High TD and LMA can enhance water stress tolerance, but can also increases the water and gas diffusion resistance of the blade, and reduce overall diffusion efficiency [10,63,64]. SD and VD can be increased to shorten water and gas transportation distances in mesophyll tissue to reduce this resistance [6,32]. In this work, VD, SD_ada_, and SD_aba_ were found to be significantly correlated with the ratio of PT/ST (Table 2). Under drought conditions, PT/ST is increased and photosynthesis per unit leaf area improves [4]. At the same time, PT/ST is known to be negatively correlated with hydraulic resistance [34]. The positive correlation of PT/ST with VD and SD indicated that *A. sparsifolia* exhibits special adaptation to reduce hydraulic resistance and enhance the conduction of CO_2_ and H_2_O in the mesophyll.

The relationship of stomatal traits on both sides of the leaves with other traits is not always consistent. The adaxial stomata of amphistomatous leaves provide a unique advantage to thick leaves (high LT and LMA) by shortening the CO_2_ transport pathway between the atmosphere and chloroplasts [65,66]. However, it also increases the risk of water loss on the adaxial surface. As a result, thick leaves need to invest a high amount of carbon to enable veins to balance water loss onthe adaxial surface [32]. However, in *A. sparsifolia*, SD_aba_ but not SD_ada_ showed a significant relationship with VD, LT, and SL_ada_ (Figure 7). As an isobilateral leaf, the upper and lower leaf structures of *A. sparsifolia* are almost symmetrical, thus the hydraulic paths on the two sides of leaves are isolated [67]. Thestomataltraits on the two sides of the leaves can be changed inconsistently [68,69] to adapt to different humidity conditions on the upper and lower leaf surfaces [70]. When natural conditions become dry and hot, isobilateral leaves preferentially close stomata on the surface that is exposed to higher irradiation (and thus reduce evaporative demand) [67,71]. Therefore, under conditions when water around the leaf is sufficient and radiation is weak, such as the conditions encountered during the morning, stomata on both sides of the leaves are completely open, providing additional CO_2_ to palisade tissues on both sides of the leaves and achieving high photosynthetic efficiency. With the increase of radiation intensity, stomata on the upper surface may be preferentially closed. Meanwhile the stomata on the lower surface remain open, and CO_2_ and H_2_O exchange continues. In this situation, the functional relationship between the lower stomata and other economic and hydraulic traits becomes closer. Therefore, the independent control of adaxial and abaxial stomata can increase the flexibly of the control of CO_2_ and H_2_Oeconomics in the mesophyll.

The LMA is a central variable of the leaf economic spectrum that captures the trade-off among leaf photosynthetic function, longevity, and structural investment [1,2,72]. In general, leaves with high LMA leaves have high LT and low N_mass_; these kinds of leaves invest a high amount of matter into structural construction and thus have low photosynthetic efficiency but improved tolerance to low fertility and drought [1,12,73]. Photosynthesis depends on an adequate of nitrogen and water. Vascular plants increase their investment in VD to enhance their water transport capacity in response to dryness, however, vein construction is considered uneconomic because it consumes large amounts of carbon [74,75]. Nitrogen directly affects vegetative growth and determines photosynthetic capacity becauseit is mostly present in photosynthetic enzymes [76].The N_mass_ of *A. sparsifolia* at all the sampling sites in this study fell in the range of 10.51–23.31 mg g^−1^, which included the average value of desert plants in Xinjiang [77] and other desert plants such as *Reaumuria soongorica* [78]. The leaves of *A. sparsifolia* were isobilateral with developed palisade tissue. Its nitrogen concentration per unit leaf area was significantly positively correlated with LMA and TD, indicating that even if the “slow return” strategy was adopted to conserve water and nutrients, nitrogen concentration per unit leaf area remained at levels sufficient for matter production.

### 4.2. Contributions of Environmental Variables on the Leaf Functional Traits of A. sparsifolia

The multidimensional variation pattern of plant functional traits is the product of plant adaptation to various environmental variables and thus reflects the net outcome of environmental filtering [79]. On the global or large spatial scale, climate is the main ecological factor that determines the distribution characteristics of plant species [80]. In this regard, *A. sparsifolia* survive only in arid areas wherein the MAP is less than 200 mm (In this work, MAP ranged from 16 mm to 166 mm across all sampling sites) (Figure 1). The limiting of MAP and nutrients provided the greatest contributions to the changes in LT and SL of *A. sparsifolia* (Figure 3, Figure 6). Compared with plants in humid and semi-arid areas [3,4], *A. sparsifolia* demonstrated significantly decreased SL and significantly increased SD. The significant correlation between SL and MAP (Figure 3) showed that climatic conditions had an important influence on stomatal morphology. As mentioned above, the variation in SL is more economical than in SD. Therefore, the SL of *A. sparsifolia* responded preferentially to the change in MAP. SAN and SOC in the soil are derived from the decomposition of litter by soil microorganisms; precipitation increase SAN and SOC by increasing microorganism activity [81]. Therefore, we inferred that with the increase in MAP, soil nutrients availability gradually increased. This increase then affected leaf traits.

In hyper-arid regions, salinization is a fundamental cause of plant water deficit [41,42,43,44]. The roots of *A. sparsifolia* can be connected to groundwater through developed roots and the damage inflicted by soil salinization can then be avoided. However, groundwater in the Taklimakan Desert is very salty [49]. SEM observation revealed that a large number of salt crystals blocked stomata (Figure S1), illustrating that *A. sparsifolia* can remove salt through its stomata. Salt ions are considered to be an essential osmotic regulator [82] that can control stomata opening and closure by regulating the osmotic potential of guard cells [83]. However, the activity of blocked pores is significantly weakened. We inferred that the number of stomata on the leaf surface might be forcibly increased for gas exchange. However, this conjecture needs further verification.

Nutrient limitation, especially nitrogen restriction, has a great effect on the leaf area of the whole plant. Reductions in leaf area may increase the irradiance of lower leaves and increase the total plant LMA [84,85]. Given that *A.* *sparsifolia* can compensate for the lack of SAN through BNF [49], the lack of SOC may become an important factor that affects plant leaf growth and LMA. Water deficiency increases the thickness of the cell wall, the content of collenchyma, and the density of veins, thus increasing mesophyll tissue density [86]. Physiological drought caused by salt stress is similar to that caused by water stress [41,42,43,84]. Water stress has an important effect on TD, VD, and LMA (Figure 4). Given that salt also can increase the size or number of mesophyll cells [87,88], it has an important impact on TD, VD, and LMA.

Temperature is the key driving force ofthe formation of soil microbial communities because it affects the carbon utilization, structure, and functions of fungi [89] and plays an essential role in the decomposition and transformation of soil nutrients [90]. The BNF is also affected by temperature, water, and salt conditions [50,52]. Consequently, temperature affects nitrogen concentration in the leaves of *A. sparsifolia*. In addition, pH might influence N_area_ through water absorption given that high soil pH associated with low water absorption by plant roots [91], likely because pH affects aquaporin function [92]. At the same time, when water shortage occurs, plants increase nitrogen distribution in their leaves, improve cell osmotic pressure, reduce water consumption by reducing stomatal conductance, and strengthen water protection in vivo [91,93,94]. Therefore, in *A. sparsifolia*, the change in leaf traits is the comprehensive response to different water, salt, and nutrients conditions in hyper-arid areas.

## 5. Conclusions

We confirmed the coupling relationship between leaf economic and hydraulic traits in a species that is widely distributed in hyper-arid regions. The economic traits, hydraulic traits, and mesophyll tissue structure of *A. sparsifolia* tended to ensure the coordination of water and nutrient conservation with CO_2_ and H_2_O diffusion in mesophyll tissue to balance matter production and investment. Leaf functional traits were affected by different environmental variables. However, this study had an important shortcoming: that is, *A. sparsifolia* can survive only in arid areas. Future studies need to select several species that can survive over a broad range of precipitation gradients to confirm further effect of precipitation on the relationship between economic and hydraulic traits.

## Figures and Tables

**Table 1:** Values and abbreviations of the economic and hydraulic traits of *Alhagi* *sparsifolia*.

	Traits Name	Abbre	Unit	Mean ± SE	CV (%)
Economic traits	Leaf dry mass per area	LMA	g m^−2^	116.4 ± 3.1	17.16
	Leaf thickness	LT	mm	0.39 ± 0.01	19.35
	Leaf tissue density	TD	g cm^−3^	0.31 ± 0.01	23.83
	Leaf nitrogen concentration per mass	N_mass_	mg g^−1^	14.60 ± 0.52	23.13
	Leaf nitrogen concentration per area	N_area_	g m^−2^	1.67 ± 0.05	20.23
Hydraulic traits	Vein density	VD	mm mm^−2^	11.49 ± 0.22	12.46
	Maximum stomatal conductance to water vapor	g_wmax_	mol m^−2^ s^−1^	0.92 ± 0.03	20.37
	Stomatal length on the adaxial surface of the leaf	SL_ada_	μm	16.46 ± 0.30	11.97
	Stomatal length on the abaxial surface of the leaf	SL_aba_	μm	16.08 ± 0.25	10.12
	Stomatal density on the adaxial surface of the leaf	SD_ada_	mm^−1^	248.4 ± 10.86	28.34
	Stomatal density on the abaxial surface of the leaf	SD_aba_	mm^−1^	300.2 ± 9.30	20.05

Notes: Abbre, abbreviations; SE, standard error; CV, coefficient of variation, this calculated as standard deviation/mean.

**Table 2:** Pearson correlation coefficients of mesophyll structure with leaf economic and hydraulic traits.

	LMA	LT	TD	N_mass_	N_area_	VD	g_wmax_	SL_ada_	SL_aba_	SD_ada_	SD_aba_
UPT	**0.37**	**0.86**	**−0.51**	−0.28	0.03	−0.05	**−0.47**	**0.51**	0.24	−0.12	**−0.60**
LPT	**0.31**	**0.70**	**−0.40**	**−0.55**	−0.30	0.07	**−0.37**	0.13	−0.18	−0.03	−0.27
PT	**0.39**	**0.76**	**−0.42**	**−0.47**	−0.14	0.09	**−0.38**	0.21	−0.07	-0.05	**−0.34**
ST	0.23	**0.63**	**−0.40**	−0.21	−0.01	−0.24	**−0.51**	0.24	0.17	**−0.36**	**−0.61**
PT/ST	0.06	−0.07	0.09	−0.13	−0.09	**0.31**	0.24	−0.09	−0.22	**0.33**	**0.36**

Notes: Significant correlations are indicated in bold. UPT, thickness of palisade parenchyma in the upper layer; LPT, thickness of palisade parenchyma in the lower layer; PT, thickness of palisade parenchyma; ST, thickness of spongy parenchyma; PT/ST, ratio of thickness of palisade parenchyma to spongy parenchyma. The abbreviations of leaf economic and hydraulic traits are shown in Table 1.

## Data Availability

All data reported here is available from the authors upon request.

## Supplementary Materials

The following are available online at https://www.mdpi.com/article/10.3390/plants10091867/s1, Table S1: Mean and SE values for traits measured for each sampling site, Table S2: Loading scores of 11 traits in the PCA, Figure S1: The SEM pictures of stomata with salt crystals. (A) Picture of the leaf 28 surface. (B) The picture of the leaf section. The red circle marked the salt crystals 29 discharged from the stomata.
