# Supplementary material for: Coupling Relationship of Leaf Economic and Hydraulic Traits of Alhagi sparsifolia Shap. in a Hyper-Arid Desert Ecosystem
Source: Plants (Basel). 2021 Sep 9;10(9):1867. doi: 10.3390/plants10091867 (PMC8465641; doi:10.3390/plants10091867)
Supplement: Supplementary file 1 [file plants-10-01867-s001.zip › plants-1357383-supplementary.pdf]

Table S1. Mean and SE values for traits measured for each sampling site.

	LMA	N_{area}	N_{mass}	LT	TD	g_{wmax}	VD	SL_{aba}	SL_{ada}	SD_{ada}	SD_{aba}
S1	128.2 ± 2.7	1.75 ± 0.10	13.67 ± 0.49	0.31 ± 0.01	0.42 ± 0.01	1.25 ± 0.15	12.81 ± 0.30	14.82 ± 0.08	14.66 ± 1.09	433.3 ± 19.2	444.4 ± 22.2
S2	106.8 ± 5.7	1.16 ± 0.07	10.84 ± 0.12	0.32 ± 0.01	0.33 ± 0.03	0.96 ± 0.02	12.04 ± 0.32	14.88 ± 1.46	15.89 ± 0.34	138.8 ± 5.5	316.7 ± 9.6
S3	109.8 ± 8.2	1.81 ± 0.14	16.45 ± 0.24	0.44 ± 0.01	0.25 ± 0.02	0.81 ± 0.03	9.91 ± 0.40	20.21 ± 0.40	19.10 ± 0.15	194.6 ± 3.2	220.1 ± 7.3
S4	100.6 ± 1.7	1.35 ± 0.03	13.45 ± 0.05	0.45 ± 0.01	0.22 ± 0.00	0.89 ± 0.05	12.61 ± 0.29	17.82 ± 0.37	14.57 ± 0.51	202.6 ± 18.4	319.0 ± 7.0
S5	146.5 ± 10.0	1.92 ± 0.10	13.16 ± 0.63	0.46 ± 0.01	0.32 ± 0.02	0.94 ± 0.02	12.36 ± 0.16	16.95 ± 0.36	16.89 ± 0.83	283.2 ± 8.3	291.9 ± 8.3
S6	125.0 ± 7.4	2.27 ± 0.17	18.16 ± 0.48	0.44 ± 0.01	0.28 ± 0.02	0.77 ± 0.06	12.14 ± 0.09	17.42 ± 0.37	16.25 ± 1.00	199.4 ± 9.7	246.9 ± 5.4
S7	99.3 + 5.2	1.71 ± 0.13	17.17 ± 0.47	0.33 ± 0.01	0.30 ± 0.03	1.24 ± 0.05	12.44 ± 0.39	16.59 ± 0.53	18.51 ± 0.45	314.2 ± 11.2	350.9±8.4
S8	130.1 ± 4.2	2.11 ± 0.05	16.24 ± 0.80	0.26 ± 0.00	0.50 ± 0.01	0.93 ± 0.06	11.82 ±0.10	17.27 ± 0.54	15.42 ± 0.40	241.5 ± 1.4	314.2 ± 15.2
S9	124.2 ± 2.6	1.30 ± 0.03	10.51 ± 0.04	0.41 ± 0.00	0.30 ± 0.01	0.980.03	12.93 ± 0.10	17.31 ± 0.08	15.55 ± 0.43	266.4 ± 7.0	328.7 ± 1.5
S10	119.8 ± 4.9	1.70 ± 0.04	14.21 ± 0.38	0.45 ± 0.01	0.27 ± 0.01	0.71 ± 0.01	9.52 ± 0.02	15.42 ±0.32	15.44 ± 0.22	277.5 ± 11.1	240.8 ± 3.2
S11	73.2 ± 7.8	1.70 ± 0.15	23.31 ± 0.30	0.26 ± 0.01	0.27 ± 0.01	1.02 ± 0.09	8.73 ± 0.04	12.48 ± 0.43	15.3 ± 0.16	255.2 ± 1.6	347.7 ± 18.0
S12	139.5 ± 3.9	1.68 ± 0.10	11.99 ± 0.47	0.48 ± 0.01	0.29 ± 0.01	0.73 ± 0.03	9.40 ± 0.09	17.44 ± 0.90	16.41 ± 0.52	191.1 ± 5.5	232.8 ± 13.1
S13	105.8 ± 2.6	1.29 ± 0.05	12.17 ± 0.21	0.45 ± 0.01	0.23 ± 0.01	0.72 ± 0.01	12.07 ± 0.06	15.25 ± 0.53	14.34 ± 0.60	215.3 ± 7.3	263.2 ± 12.7
S14	121.3 ± 2.4	1.59 ± 0.04	13.12 ± 0.01	0.40 ± 0.01	0.31 ± 0.01	0.93 ± 0.02	12.04 ± 0.08	16.56 ± 0.39	16.78 ± 0.72	264.8 ± 5.8	292.3 ± 5.9

Note: SE: standard error; the abbreviations and the unit of traits showed in Table 1; S1–S14: the sampling sites marked in the Figure 1.

Table S2. Loading scores of 11 traits in the PCA.

	PC1	PC2	PC3	PC4
eigenvalue	3.66	2.24	2.00	1.25
SD_{aba}	0.49	−0.03	0.16	0.09
g_{wmax}	0.45	−0.06	−0.02	0.42
LT	−0.44	−0.16	0.11	0.09
SD_{ada}	0.36	−0.18	−0.03	0.00
TD	0.31	−0.35	−0.18	−0.30
SL_{ada}	−0.26	−0.36	−0.16	0.35
VD	0.16	−0.37	0.36	0.21
N_{mass}	0.15	0.37	−0.51	−0.01
LMA	−0.10	−0.60	−0.09	−0.24
SL_{aba}	−0.08	−0.07	−0.37	0.67
N_{area}	0.03	−0.22	−0.61	−0.22

Note: traits are arranged in descending order of their loading scores on the PC1. The abbreviations of leaf economic and hydraulic traits are shown in Table 1.

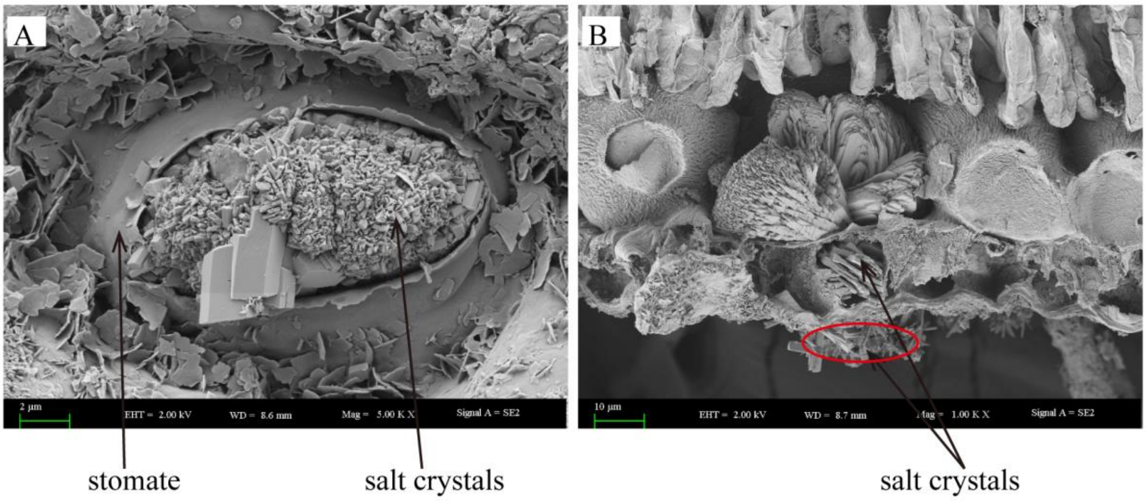

Figure S1. The SEM picutres of stomata with salt crystals. (**A**). picture of the leaf surface. (**B**). The picture of the leaf section. The red circle marked the salt crystals discharged from the stomata.
